# Association between social capital and loneliness among older adults: a cross-sectional study in Anhui Province, China

**DOI:** 10.1186/s12877-020-01973-2

**Published:** 2021-01-07

**Authors:** Zhongliang Bai, Zijing Wang, Tiantai Shao, Xia Qin, Zhi Hu

**Affiliations:** 1 Department of Epidemiology and Biostatistics, School of Public Health, Anhui Medical University, Hefei, 230032 Anhui China; 2 Department of Health Services Management, School of Health Services Management, Anhui Medical University, Hefei, 230032 Anhui China

**Keywords:** Social capital, Aging, Health, Loneliness, China

## Abstract

**Background:**

We aimed to examine the association between social capital and loneliness in Anhui Province, China.

**Methods:**

Data were collected from a cross-sectional study using a multi-stage stratified cluster sampling strategy. Data on demographic characteristics, socioeconomic factors, social capital, and loneliness in 1810 older adults (aged 60 years and older) were used for analysis. Binary logistic regression models and a classification and regression tree model were performed to assess the association of social capital and loneliness.

**Results:**

Our results indicated that social capital in terms of lower level of social participation (AOR = 1.38; 95% CI: 1.10–1.74), social connection (AOR = 1.51; 95% CI: 1.18–1.93), and reciprocity (AOR = 1.47; 95% CI: 1.13–1.90) were associated with higher odds of developing loneliness. We noted the interactive effect of different social capital dimensions on loneliness, suggesting that the risk for suffering loneliness was greatest in older people limited in functional ability, with less trust, less social connection, and less social participation.

**Conclusions:**

Our findings show that social capital is associated with loneliness in older adults. This implies that social capital, especially in terms of trust, social connection, and social participation may be significant for alleviating loneliness in later life.

## Background

Although the size of an aging society among the people has been soaring globally, yet a prolonged life is not accompanied by good health and well-being [1, 2]. Studies have found that loneliness, as a negative emotional status, can degenerate the health and well-being of older people during aging [3–5]. For example, loneliness has been found to prompt coronary heart disease and stroke [6], and dementia [7], consequently leading to poor quality of life [8, 9].

Loneliness is frequent among older people [5], yet the prevalence of loneliness varies across different countries according to different assessment tools and study samples. A national longitudinal study in Singapore found that about 23.0% of older people suffered from chronic loneliness [5]. A cross-sectional survey in Finland found that 27.3% of the older adults had frequent loneliness [10]. Prior findings from the Chinese Longitudinal Healthy Longevity Survey (CLHLS) revealed that about 22.9% of older men and 30.6% of older women experienced loneliness [11]. Therefore, how to prevent and reduce the incidence of loneliness is a pressing issue in public health research.

A previous study has evidenced that determining the risk factors of loneliness is of importance to prevent loneliness [12]. Some factors such as females [11], advanced age [13], low level of educational attainment, and socioeconomic status [14], residing in rural areas [15] have been identified as having a greater odds of developing loneliness in later life. Additional factors including physical inactivity, living alone, retirement, and loss of companions or friends were associated with a higher risk of loneliness among older individuals [16–19]. In the last decades, an increasing volume of studies concluded that more engagement in activities and good relationships with social contact could benefit older adults and reduce loneliness [14, 20]. With the development of determinants of health, the significance and relevancy of social resources, including social capital, in maintaining the well-being and health of older people have been emphasized [13]. Social capital refers to a concept that describing social relations at individual or community levels that can be obtained through mutual interaction within a neighborhood, community, or society [10, 21, 22]. Growing studies have documented that social capital is related to the health and wellness of older people [23, 24], and many studies including from China have examined the association of social capital and loneliness [10, 14, 15].

However, existing researches exploring the relationship between social capital and loneliness among older populations assessed social capital with varied factors and obtained mixed findings. For example, a study in Western Finland found that low levels of social capital that measured by social contacts, participation, trust, and sense of belonging, while only lower trust was associated with loneliness among the oldest age group [10]. In a study conducted in 14 European countries, social capital, including regular social participation was linked to a decreased experience of loneliness and a reduction in the impact of loneliness in low-income families [14]. A prior study found that social capital (bonding social capital and bridging social capital) was associated with loneliness among rural widowed older people in China [15]. Similar conclusions have also been drawn from an intervention study in Spain, which indicated that the risk of experiencing loneliness decreased with an increase in social capital regarding social participation and social support [25]. Hence, to better explore and examine the linkage of social capital and loneliness among older adults, an accepted and defined method to measure social capital is highly needed.

As mentioned above, loneliness in later life was exposed to a myriad of factors, including a low level of social capital [11, 13–15, 19]. Despite that these factors had been well explored in many studies, whether these factors and social capital interact to impact loneliness remains to be confirmed by further studies. Collectively, a more accurate and comprehensive analytical approach exploring the interaction of different variables that prompt health outcomes in the older population is proposed [26].. Exploring multiple interactions is fundamental in obtaining the most targeted and effectual interventions to reduce the onset of loneliness and these findings may be valuable in developing appropriate intervention measures or programs to prevent the incidence of loneliness.

Given this, the present study aims to examine the relationship between social capital (six dimensions) and loneliness and investigates whether there is an interactive effect of social capital and demographic factors and health-related factors on loneliness among community-dwelling older adults in Anhui Province, China.

## Methods

### Study design and data collection

This cross-sectional study was carried out from July to September 2017 in Anhui Province, which is located in the east of China (Additional file 1). As of December 2019, the population residing in Anhui is around 71.19 million, among which people aged 60 years and above had accounted for about 18.41% [27]. In brief, 1935 participants were interviewed, while 1810 were finally included for analysis. A detailed description of the study design and data collection has been fully described previously [28, 29].

### Measures

#### Measurement of loneliness

Loneliness, the main outcome variable, was assessed by a single question in line with previous studies [10, 11, 30]. Participants were asked the question “Do you have the feeling of loneliness?” with answers ranging from “often, sometimes, and never”. We dichotomized this variable into two categories (yes, no) by combining “often and sometimes” as the presence of loneliness, while “never” as the absence of loneliness, which was in line with other studies [10, 11, 31]. Details about the measurement of loneliness please refer to Additional file 2.

#### Measurement of social capital

Social capital, the main independent variable, included six dimensions: social participation, social connection, social support, trust, cohesion, and reciprocity. Social capital was measured with a five-point Likert scale and 22 items. More information about the measurement of social capital has been published previously [28, 29] and can also be found in Additional file 2.

Subjects were asked to respond to each item with answers ranging from 1 = “never”, 2 = “seldom”, 3 = “usually”, 4 = “often”, and 5 = “more often”, where each number indicated the associated score accordingly. The score ranges for each dimension were obtained by summarizing answers to each item as follows; social participation (4–20), social support (4–20), social connection (3–15), trust (3–15), cohesion (5–25), reciprocity (3–15). A higher score denoted a better social capital. During analysis, binary variables (high and low level) were generated by dichotomizing each dimension into two categories according to their relative median value, which was in accordance with other studies [32, 33].

#### Measurement of other variables

Other variables including age, sex, body mass index (BMI, kg/m^2^), living status, residence (urban, rural), marital status, education level, functional ability, smoking status, drinking status, and the number of diseases were collected as well. In this study, we measured the functional ability of the participants using the work of Lawton and Brody [28, 34], meanwhile, the number of diseases was collected based on the physical health section of the Older Americans Resources and Services (ORAS) [35], which were ranked into four categories: 0, 1, 2, > 2 [36]. More detailed information about these variables has been described in our previous papers [28, 29].

### Statistical analysis

First, to compare the difference between two groups (lonely versus not lonely), a Chi-square test was used with variables expressed as numbers and percentages.

Second, we applied logistic regression models to calculate the odds ratio (OR) of developing loneliness and its 95% confidence interval (95% CI). Based on the literature review [23, 37], related variables such as age, sex, BMI, living status, residence, marital status, education level, functional ability, number of diseases, smoking status, and drinking status, were adjusted in all regression models.

Third, we used a classification and regression tree (CART) model to explore the interactive relationship between some indicators of social capital and demographic factors and health-related factors associated with loneliness. This nonparametric model has been used to explore the different interactions among various variables in public health research [38, 39]. Several homogenous subgroups related to the development of loneliness were generated using this model. The variables included in this model were based on the unadjusted results from the logistic regression model. The tree model growing method has been described previously [28].

There no missing data in this study and all analyses were processed in the SPSS 23.0 statistical software (SPSS Inc., Chicago, IL, USA). The *P*-value that smaller than 0.05 indicated the statistical significance level. We used the ArcMap software (version 10.6) to make the map to show the location of study area. The map is available on request from the corresponding author.

## Results

### Results of descriptive analysis

The summary statistics of the general characteristics of the respondents are presented in Table 1. The study included 1810 participants (71.20 ± 7.51 years). There were differences in age, sex, BMI, living status, residence, marital status, education level, functional ability, number of diseases, drinking status, and social capital dimensions between the two groups (lonely versus not lonely). About 38.7% (700 out of 1810) of the participants reported a sense of loneliness in this study, of which 39.7% (278/700) were between 60 and 69 years, 62.3% (436/700) were females, 48.7% (341/700) of subjects’ BMI was between 18.5–22.9, 81.0% (567/700) were living with others, about 65.0% (455/700) lived in the rural area, 67.9% (475/700) belonged to married or cohabited group, and 82.0% (574/700) attended primary school and below. Meanwhile, above half the lonely participants 64.9% (454/700) reported limited functional ability. 38.1% (267/700) of them suffered at least one disease. Most of the participants reporting loneliness were non-smoker (80.3%) and non-drinker (84.9%).

**Table 1:** General characteristics of the respondents (*N* = 1810)

	Loneliness			
Variables	No *N* = 1110	Yes *N* = 700	χ^2^	*p*-value
**Age (years)**			37.365	< 0.001
60–69	545 (49.1%)	278 (39.7%)		
70–79	435 (39.2%)	268 (38.3%)		
≥ 80	130 (11.7%)	154 (22.0%)		
**Sex**			10.881	0.001
Male	506 (45.6%)	264 (37.7%)		
Female	604 (54.4%)	436 (62.3%)		
**BMI (kg/m**^**2**^**)**			8.722	0.033
< 18.5	108 (9.7%)	81 (11.6%)		
18.5–22.9	484 (43.6%)	341 (48.7%)		
23.0–27.4	417 (37.6%)	227 (32.4%)		
≥ 27.5	101 (9.1%)	51 (7.3%)		
**Living status**			30.518	< 0.001
Not living alone	1000 (90.1%)	567 (81.0%)		
Living alone	110 (9.9%)	133 (19.0%)		
**Residence**				< 0.001
Urban	556 (50.1%)	245 (35.0%)		
Rural	554 (49.9%)	455 (65.0%)		
**Marital status**			60.266	< 0.001
Married/cohabited	927 (83.5%)	475 (67.9%)		
Single	183 (16.5%)	225 (32.1%)		
**Education level**			63.648	< 0.001
Primary school and below	717 (64.6%)	574 (82.0%)		
Junior school	219 (19.7%)	72 (10.3%)		
High school and above	174 (15.7%)	54 (7.7%)		
**Functional ability**			222.842	< 0.001
Robust	786 (70.8%)	246 (35.1%)		
Limited	324 (29.2%)	454 (64.9%)		
**Number of diseases**			67.128	< 0.001
0	378 (34.1%)	152 (21.7%)		
1	447 (40.3%)	267 (38.1%)		
2	197 (17.7%)	150 (21.4%)		
> 2	88 (7.9%)	131 (18.7%)		
**Smoking status**			3.848	0.146
Non-smoker	850 (76.6%)	562 (80.3%)		
Former smoker	62 (5.6%)	37 (5.3%)		
Smoker	198 (17.8%)	101 (14.4%)		
**Drinking status**			7.737	0.021
Non-drinker	890 (80.2%)	594 (84.9%)		
Former drinker	43 (3.9%)	27 (3.9%)		
Drinker	177 (15.9%)	79 (11.3%)		
**Social participation**			40.766	< 0.001
High	705 (63.5%)	338 (48.3%)		
Low	405 (36.5%)	362 (51.7%)		
**Social support**			4.670	0.031
High	578 (52.1%)	328 (46.9%)		
Low	532 (47.9%)	372 (53.1%)		
**Social connection**			67.738	< 0.001
High	862 (77.7%)	417 (59.6%)		
Low	248 (22.3%)	283 (40.4%)		
**Trust**			37.188	< 0.001
High	690 (62.2%)	333 (47.6%)		
Low	420 (37.8%)	367 (52.4%)		
**Cohesion**			37.064	< 0.001
High	723 (65.1%)	355 (50.7%)		
Low	387 (34.9%)	345 (49.3%)		
**Reciprocity**			53.083	< 0.001
High	675 (60.8%)	303 (43.3%)		
Low	435 (39.2%)	397 (56.7%)		

### Results of logistic regression analysis

Table 2 shows the results of the logistic regression models. In model 1 (unadjusted), subjects aged ≥80 years (OR = 2.32; 95% CI: 1.76–3.06), who were females (OR = 1.38; 95% CI: 1.14–1.68), living alone (OR = 2.13; 95% CI: 1.62–2.80), residing in rural areas (OR = 1.86; 95% CI: 1.53–2.26), were single (OR = 2.40; 95% CI: 1.92–3.00), with low education level (OR = 2.58; 95% CI: 1.86–3.57), and limited functional ability (OR = 4.48; 95% CI: 3.66–5.48) were found to have a higher risk for experiencing loneliness. The odds for developing loneliness increased with the number of diseases and were the highest among respondents reporting ≥2 diseases (OR = 3.70; 95% CI: 2.66–5.15). Individuals with lower social participation (OR = 1.86; 95% CI: 1.54–2.26), social support (OR = 1.23; 95% CI: 1.02–1.49), social connection (OR = 2.36; 95% CI: 1.92–2.90), trust (OR = 1.81; 95% CI:1.49–2.19), cohesion (OR = 1.82; 95% CI: 1.50–2.20), and reciprocity (OR = 2.03; 95% CI: 1.68–2.46) were at a greater risk of developing loneliness.

**Table 2:** Logistic regression analysis examining the association between social capital and loneliness (N = 1810)

Variables	Model 1	Model 2
	**OR (95% CI)**	**AOR (95% CI)**
**Age (years)**		
60–69	1	1
70–79	1.21 (0.98–1.49)	0.96 (0.75–1.22)
≥ 80	2.32 (1.76–3.06) ***	1.25 (0.90–1.74)
**Sex**		
Male	1	1
Female	1.38 (1.14–1.68) **	1.05 (0.79–1.38)
**Body mass index (kg/m**^**2**^**)**		
Normal weight	1	1
Underweight	1.06 (0.77–1.47)	0.81 (0.56–1.17)
Overweight	0.77 (0.62–0.96) *	0.88 (0.69–1.12)
Obese	0.72 (0.50–1.03)	0.68 (0.45–1.03)
**Living status**		
Not living alone	1	1
Living alone	2.13 (1.62–2.80) ***	1.39 (0.96–2.00)
**Residence**		
Urban	1	1
Rural	1.86 (1.53–2.26) ***	1.26 (1.00–1.60)
**Marital status**		
Married/cohabited	1	1
Single	2.40 (1.92–3.00) ***	1.66 (1.22–2.25) **
**Education level**		
High school and above	1	1
Primary school and below	2.58 (1.86–3.57) ***	1.10 (0.76–1.60)
Junior school	1.06 (0.71–1.59)	0.80 (0.51–1.24)
**Functional ability**		
Robust	1	1
Limited	4.48 (3.66–5.48) ***	3.10 (2.45–3.93) ***
**Number of diseases**		
0	1	1
1	1.49 (1.17–1.89) **	1.30 (0.99–1.70)
2	1.89 (1.43–2.51) ***	1.48 (1.08–2.03) *
> 2	3.70 (2.66–5.15) ***	2.38 (1.64–3.45) ***
**Smoking status**		
Non-smoking	1	1
Former smoking	0.90 (0.59–1.37)	1.23 (0.72–2.11)
Smoking	0.77 (0.59–1.00)	1.14 (0.80–1.62)
**Drinking status**		
Non-drinking	1	1
Former drinking	0.94 (0.58–1.54)	0.81 (0.44–1.49)
Drinking	0.67 (0.50–0.89) *	0.80 (0.56–1.13)
**Social participation**		
High	1	1
Low	1.86 (1.54–2.26) ***	1.38 (1.10–1.74) **
**Social support**		
High	1	1
Low	1.23 (1.02–1.49) *	0.79 (0.62–1.01)
**Social connection**		
High	1	1
Low	2.36 (1.92–2.90) ***	1.51 (1.18–1.93) **
**Trust**		
High	1	1
Low	1.81 (1.49–2.19) ***	1.21 (0.93–1.56)
**Cohesion**		
High	1	1
Low	1.82 (1.50–2.20) ***	1.15 (0.88–1.49)
**Reciprocity**		
High	1	1
Low	2.03 (1.68–2.46) ***	1.47 (1.13–1.90) **

Note: Model 2 adjusted age, sex, body mass index, living status, residence, marital status, education level, functional ability, number of diseases, smoking, and drinking status

OR: odds ratio, 95% CI: 95% confidence interval

*p < 0.05; **p < 0.01; ***p < 0.001

After adjusting all covariates in model 2, respondents with lower social participation (AOR = 1.38; 95% CI: 1.10–1.74), social connection (AOR = 1.51; 95% CI: 1.18–1.93), and reciprocity (AOR = 1.47; 95% CI: 1.13–1.90) were more likely to suffer from loneliness.

### Results of classification and regression tree model

**Fig. 1:**
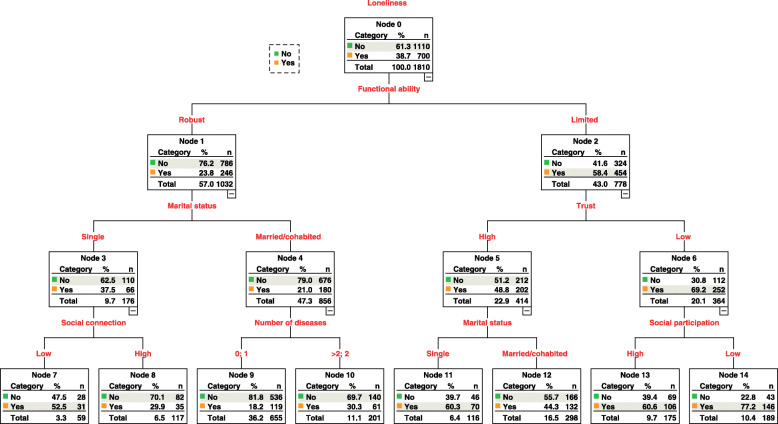
CART model analysis examing the interactive association between social capital and loneliness. (This classification and regression tree shows the variables related to the development of loneliness and the likelihood of experiencing loneliness in the node boxes, which also displays the interactive relationship between social capital and demographic factors and health-related factors associated with loneliness)

The results of the CART model are shown in Fig. 1. The development of loneliness was associated with functional ability, marital status, trust, social connection, number of diseases, and social participation. The interactive effect among social capital dimensions and diverse variables were also observed.

Our analyses indicated that functional ability was the most determinant factor related to loneliness. The sample was therefore split into subsets based on this factor. Participants with a robust functional ability (Node 1), married or cohabited (Node 4), and reported no or less number of diseases (Node 9), were least likely to experience loneliness. However, participants who were single (Node 3) and with a low level of social connection (Node 7) were more prone to experience loneliness as compared to those with a higher level of social connection (Node 8).

Those who reported limitations in functional ability (Node 2) and had a lower level of trust (Node 6) and social participation (Node 14) were the most likely to experience loneliness. Meanwhile, subjects with a higher level of trust (Node 5) and were single (Node 11), inclined to suffer from loneliness than those who were married or cohabited (Node 12).

## Discussion

In this study, we examined the association between social capital and loneliness among the older population and explored the interactive effect of social capital and demographic factors and health-related factors on loneliness in Anhui Province, China. Our results confirmed the association of social capital and loneliness and the combined influence of social capital and some other factors on the development of loneliness in later life, suggesting the relevance of social capital in preserving the emotional health of older people.

The results of the current study demonstrated the relationship between loneliness and social capital. Specifically, older people who lacked social capital concerning social participation, social connection, and reciprocity were more likely to experience loneliness, which was echoed by findings from previous studies [10, 20, 25, 40]. Similar to our results, Nyqvist et al. [10] found that infrequent social connection with neighbours had an increased likelihood of being lonely among older people aged 65–80 years in Western Finland. Results from the fifth wave of the Survey of Health, Ageing and Retirement in Europe (SHARE) also concluded that more social participation was a protective factor for loneliness and mitigated the impact of unfavorable socioeconomic status among older people [14]. Some studies also suggested that forming and building reciprocal connections or relationships with others could alleviate the impact of some mental health issues, including loneliness [20, 41].

According to the results of our adjusted logistic regression analysis, a non-significant association was observed between a lower level of social capital regarding social support, trust, and cohesion, and loneliness. Different from our results, a prior study revealed that less trust and a weak sense of belonging to the community were significantly linked to a higher risk for developing loneliness [10]. Meanwhile, a study also found that an increase of trust at the community-level also contributed to a reduction in loneliness among community-dwelling older people [42]. Also, a previous study showed that insufficient social support is significantly related to the onset of loneliness [43–45]. There are two possible reasons for this inconsistency. First, this may due to different measurements that were used to assess social capital in the current study and earlier works. For example, in this paper, social support was measured by asking participants “how often can they get mental or material support when they are in need”. But Chen et al. [43] measured social support by asking surveyed subjects the quantity of social support offered by friends. The lack of common and widely used social capital measurement tool has been recognized as a repeated issue, which contributes to some inconclusive results in this research field. Another reason may be that our adjusted model included several variables such as demographic and health-related as covariates, which were associated with social capital and loneliness. As a result, the association between social support, trust, and cohesion with loneliness was attenuated or even became non-significant. However, more research is still warranted to further verify our results in the future.

Previous studies demonstrated that limitation in functional ability, being single, and multimorbidity was related to a higher risk for developing loneliness among older people [19, 46]. In the present study, most importantly, an interacting relationship between social capital and functional ability, marital status, and the number of diseases was observed. That is, older people who reported limitations in functional ability and had a lower level of trust and social participation were the most likely to experience loneliness. This indicates that trust and social participation as a social capital dimension may be of relevance in loneliness prevention [10, 25]. Besides, single older people, who had less social connection were more prone to loneliness as compared to those who had a higher-level social connection, which further highlights the protective role of social connection in preventing the incidence of loneliness [47]. Interestingly, the significance of trust was not found in the adjusted logistic regression model, however, it was observed in the CART model. We suggest that the role of trust may depend on the appearance of other social capital dimensions and variables, which emphasizes the effectiveness of the CART model in examining the complex interactions among multiple variables that may be overlooked in the conventional analytical approach [26]. The importance of this finding lies in adding a scientific explanation of using the CART model to help examine the association between social capital and loneliness while revealing how social capital interacts with other factors and produces an effect on the development of loneliness. Additionally, the CART model was used as a predictive model to estimate the subsets of older people that are more likely to become lonely.

Regarding the assessment of loneliness, different tools and methods were employed to identify loneliness as well. For instance, the UCLA loneliness scale was the most common and validated scale used to collect loneliness data in previous research [48, 49]. However, a single item by asking participants how often they feel lonely, which potential responses included often, sometimes, and never, etc., also had been validated a good predictive validity in many studies [10, 11, 31]. Moreover, the use of a single question to assess loneliness has been proved to have relevant edges such as succinctness, easily understood, and well accepted by subjects [30]. Besides, in the process of data analysis, many studies dichotomized the status of loneliness into two groups (lonely, not lonely) by combining often and sometimes as the presence of loneliness, while rarely or never as the absence of loneliness [10, 11, 31, 37]. Despite such dichotomization could result in some loss of information about the outcome, to obtain statistical power during statistical process and make comparisons with other research. Likewise, we also categorized loneliness into two kinds in this paper.

Loneliness is a subjective and negative feeling that may be the consequence of dissatisfaction with an individual’s social relations and unsatisfied social needs [8]. Therefore, by looking at these findings from the present study, some relevant suggestions for the interventions of social capital to reduce the level and alleviate the impact of loneliness among community-dwelling older adults can be offered. First, we suggest older people should engage in more social activities, for instance, older adults are encouraged to participate the formal/informal groups (political/non-political parties, elections, hobby groups, etc.), voluntary activities, and services (heath lecture, culture, and physical education activities) within the community, which provide opportunities for them to meet the social needs, share life experiences and exchange interests. Second, the children, relatives, friends, and neighbors are encouraged to have more frequent interactions and communications with the older people and care about them, which is beneficial to maintain a good social relationship, promote and improve the quality of social connections, in turn, reduce the chance of developing loneliness among older adults. Third, to have an ideal reciprocal relationship, programs, and activities designed to cultivate and escalate the willingness to actively provide help to each other, including their relatives, friends or neighbors, and strangers, should be introduced. Lastly, to make good use of the role of social capital in the prevention of loneliness and maintain good emotional health, more attention should be paid to those who reported limitations in functional ability, had a lower level of trust and insufficient social participation, and single older people who had a less social connection.

This study has several limitations. First, since it was a cross-sectional study, which limited to conclude the causal relations between social capital and loneliness. Future studies using a longitudinal or randomized control trial design are warranted. Second, data in our study were based on self-report and might be subject to a recall or reporting bias. Nevertheless, to improve the data accuracy, we formulated clear and precise questions and carried out a pilot study before investigation. During data collection, forward or backward recall techniques were also used. Third, since our study was only conducted in Anhui Province, this might constrain the generalization of our findings to other regions or countries. Future studies that include extensive sites and larger samples are needed. Fourth, in the present study, we did not focus on the concept of emotional and social loneliness yet took loneliness as an umbrella concept instead. Besides, the variables included in this study were not broad enough; some other variables such as depression and other mental health data were not well considered. More attention should be given to these mental health factors in the future.

Despite the limitations, this study also has some strength. Findings from this study are reliable because the sample was representative and had high response rates from participants. We also used a validated and standardized social capital scale, which may facilitate the development of social capital theory. Moreover, to our knowledge, this study is the first using the CART model allowed for further exploring multi-variable interactions and yielded a straightforward and visible tree, which is suitable in devising more specific and accurate strategies to counteract the impact of loneliness.

## Conclusions

In summary, we observed an association between social capital and loneliness among the older population. Specifically, social capital in terms of social participation, social connection, trust, and reciprocity may be significant in designing intervention programs and measures to prevent and reduce the incidence of loneliness.

## Supplementary Information

**Additional file 1.** The location of sampling areas (Red areas) in Anhui province, China.

**Additional file 2.** The Questionnaire of this study (English version).

## Data Availability

The datasets generated during and/or analyzed during the current study are available from the corresponding author on reasonable request.

## Abbreviations

CLHLS: The Chinese Longitudinal Healthy Longevity Survey
BMI: Body Mass Index
ADL: The Activities of Daily Living
IADL: Instrumental Activities of Daily Living
ORAS: The Older Americans Resources and Services
CHD: Coronary Heart Disease
OR: Odds Ratio
95% CI: 95% confidence interval
CART: Classification and Regression Tree
