# Supplementary material for: Association between social capital and loneliness among older adults: a cross-sectional study in Anhui Province, China
Source: BMC Geriatr. 2021 Jan 7;21:26. doi: 10.1186/s12877-020-01973-2 (PMC7791664; doi:10.1186/s12877-020-01973-2)
Supplement: Supplementary file 1 — Additional file 1. The location of sampling areas (Red areas) in Anhui province, China. [file 12877_2020_1973_MOESM1_ESM.docx]

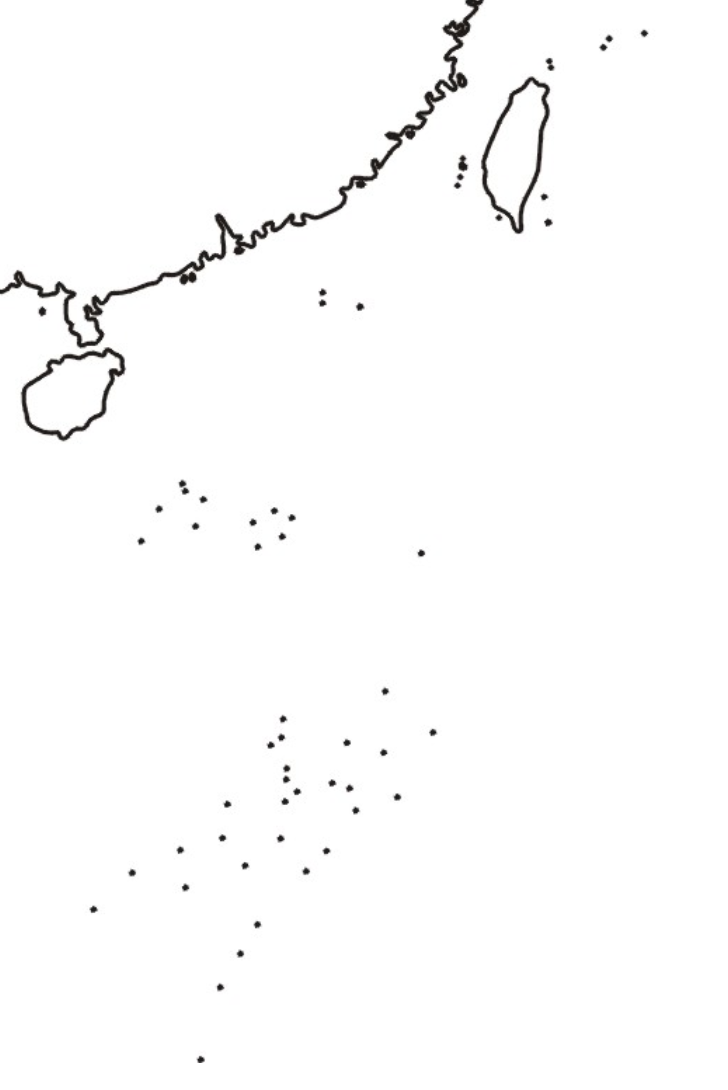

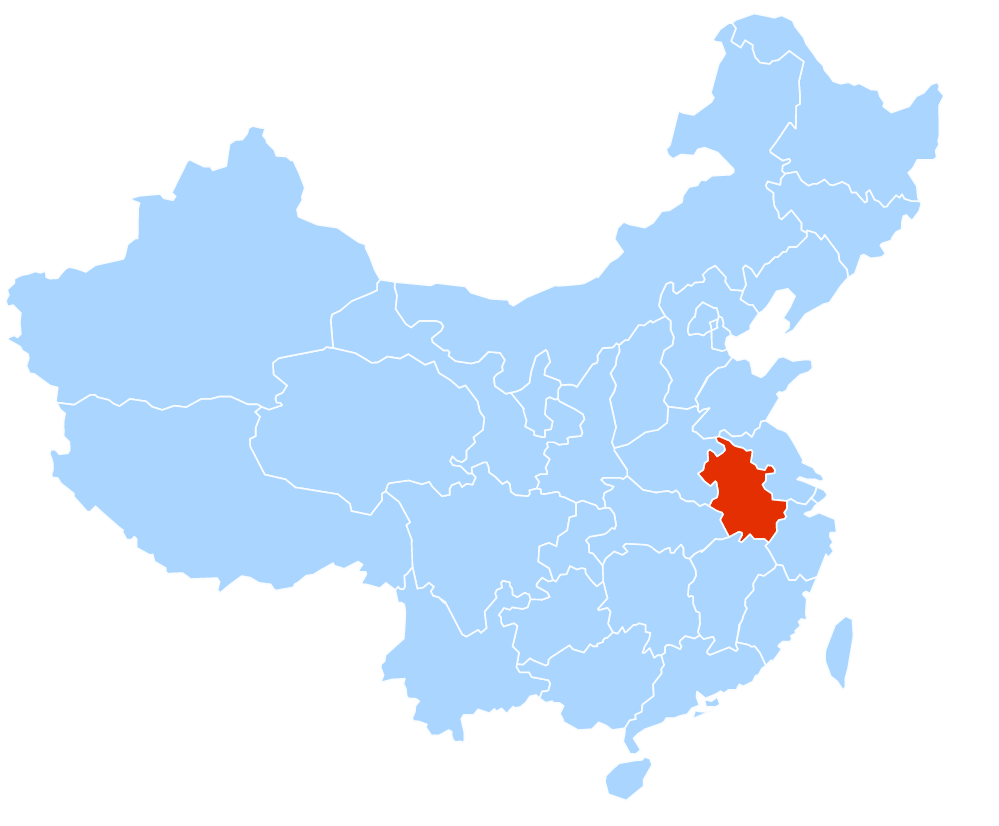


**Anhui Province**


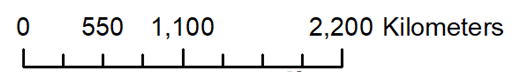


**Fig S1**. The location of sampling areas (Red areas) in Anhui Province, China. (This map shows that Anhui Province locates in the east of China and three sampling areas selected in this study; The map is available on request from the corresponding author.)
