# Supplementary material for: Association between social capital and loneliness among older adults: a cross-sectional study in Anhui Province, China
Source: BMC Geriatr. 2021 Jan 7;21:26. doi: 10.1186/s12877-020-01973-2 (PMC7791664; doi:10.1186/s12877-020-01973-2)
Supplement: Supplementary file 2 — Additional file 2. The Questionnaire of this study (English version). [file 12877_2020_1973_MOESM2_ESM.docx]

***Measurement of Social Capital***

| **Social Participation** | In the past 12 months, how often would you participate in formal groups (party or democratic parties’ elections, etc.)? | ① Never ②Seldom ③Usually ④Often ⑤More Often |
| --- | --- | --- |
|  | In the past 12 months, how often would you participate in informal groups (square dance, interest clubs, etc.)? | ① Never ②Seldom ③Usually ④Often ⑤More Often |
|  | In the past 12 months, how often would you be community volunteer (coordinator, corridor manager, etc.)? | ① Never ②Seldom ③Usually ④Often ⑤More Often |
|  | In the past 12 months, how often would you take part in community services (health lecture, cultural activities etc.)? | ① Never ②Seldom ③Usually ④Often ⑤More Often |
| **Social Support** | When you are in trouble, is there someone that provides you with mental support (i.e., comfort you)? | ① Never ②Seldom ③Usually ④Often ⑤More Often |
|  | When you are in trouble, is there someone that provides you with material support (i.e., lend you money)? | ① Never ②Seldom ③Usually ④Often ⑤More Often |
|  | When you are in trouble, are there any formal or informal groups that provide you with mental support (i.e., comfort you)? | ① Never ②Seldom ③Usually ④Often ⑤More Often |
|  | When you are in trouble, are there any formal or informal groups that provide you with material support (i.e., lend you money)? | ① Never ②Seldom ③Usually ④Often ⑤More Often |
| **Social Connection** | How often do you contact with your children? | ① Never ②Seldom ③Usually ④Often ⑤More Often |
|  | How often do you contact with your relatives? | ① Never ②Seldom ③Usually ④Often ⑤More Often |
|  | How often do you contact with your friends/ neighbors? | ① Never ②Seldom ③Usually ④Often ⑤More Often |
| **Trust** | Do you trust in your family members? | ① Never ②Seldom ③Usually ④Often ⑤More Often |
|  | Do you trust in your friends? | ① Never ②Seldom ③Usually ④Often ⑤More Often |
|  | Do you trust in someone who lives within one community/ village? | ① Never ②Seldom ③Usually ④Often ⑤More Often |
| **Cohesion** | Do you care about what happened in your community/village? | ① Never ②Seldom ③Usually ④Often ⑤More Often |
|  | Do you think the community/village is more harmonious? | ① Never ②Seldom ③Usually ④Often ⑤More Often |
|  | Do you like the community/village you live now? | ① Never ②Seldom ③Usually ④Often ⑤More Often |
|  | Do you have a feeling of being in the community/village? | ① Never ②Seldom ③Usually ④Often ⑤More Often |
|  | Do you feel reluctant, if you have to move away from the community lived now? | ① Never ②Seldom ③Usually ④Often ⑤More Often |
| **Reciprocity** | When your relatives are in trouble, will you provide help to them? | ① Never ②Seldom ③Usually ④Often ⑤More Often |
|  | When your friends/ neighbors are in trouble, will you provide help to them? | ① Never ②Seldom ③Usually ④Often ⑤More Often |
|  | When some strangers are in trouble, will you provide help to them? | ① Never ②Seldom ③Usually ④Often ⑤More Often |

***Assessment of Loneliness***

| **Loneliness** | Do you have the feeling of loneliness? | ① Often ② Sometimes ③ Never |
| --- | --- | --- |
